# Preoperative pericardial hematoma in patients with acute type A aortic dissection (AAAD): Do we need an adjusted treatment?

**DOI:** 10.1186/s13019-023-02152-1

**Published:** 2023-02-09

**Authors:** Tim Kaufeld, Beckmann Erik, Rudolph Linda, Krüger Heike, Natanov Ruslan, Arar Morsi, Korte Wilhelm, Schilling Tobias, Haverich Axel, Martens Axel, Shrestha Malakh

**Affiliations:** 1 Department of Cardiothoracic, Transplant and Vascular Surgery, Hanover Medical School, Carl – Neuberg Str.1, 30625 Hannover, Germany; 2 Minneapolis Heart Institute, Abbott Northwester Hospital, 920E 28th St., Minneapolis, MN 55417 USA; 3 Cardiovascular Medicine, Mayo Clinic, 200 First St. SW, Rochester, MN 55905 USA

## Abstract

**Objective:**

An acute type A aortic dissection (AAAD) is a critical emergency and remains one of the most challenging diseases in cardiothoracic surgery. The existence of a pericardial hematoma caused by an aortic rupture can dramatically reduce the chances of survival (Jerzewski and Kulik in J Card Surg 29(4):529–530, 2014; Mehta et al. in Circulation 105(2):200–206, 2002; Gilon et al. in Am J Cardiol 103(7):1029–1031, 2009; Isselbacher et al. in Circulation 90(5):2375–2378, 1994). We assessed the surgical outcome of a high-risk group of patients with AAAD and a pericardial hematoma.

**Methods:**

In this study we included 430 Patients (67% male; median age: 64 years) who received surgical treatment between January 2000 and January 2018 at our facility for acute aortic dissection DeBakey type I. We divided the cohort in two groups: Group A consisted of high-risk patients with a pericardial hematoma (n = 162) and Group B of patients without pericardial hematoma (n = 268).

**Results:**

Patients with a preoperative pericardial hematoma had a significantly higher requirement for preoperative mechanical resuscitation (A: 21%; B: 1.5%; *P*: < 0.001) and were relevantly more frequently admitted to the operation theater with an intubated status (A: 19.8%; B: 8.6%; *P*: < 0.001). The incidence of visceral malperfusion differed significantly between both groups (A. 11.7%, B. 6:0%; *P*: 0.034). Limited aortic arch repair (proximal aortic arch replacement) was preferred in the high-risk group (A: 51.9%; B: 40.3%; *P*: 0.020). However, survival time was generally reduced in these patients (A: 7.5 y; B: 9.9 y).

**Conclusion:**

AAAD patients with preoperative pericardial hematoma present themselves in potentially lethal conditions, with a significantly higher rate of visceral malperfusion. Despite the existence of this risk factor, a limited arch repair was favored. We have proven that cardiac compression is associated with preoperative intubation and mechanical resuscitation. Patients with pericardial hematoma must be further evaluated for preoperative pericardial drainage. In the event of long transfer times to an aortic center a slow drainage should be discussed to prevent early mortality.

## Introduction

An acute type A aortic dissection (AAAD) is a critical emergency and remains one of the most challenging diseases in cardiothoracic surgery. Without early-stage surgical intervention, it is often associated with a high mortality rate. The dissection denotes the presence of an intimal tear inside the aortic wall, which leads to a separation of the aortic layers and the consequence of further malperfusion or fatal rupturing. Malperfusion syndrome can occur in the coronary, cerebral, spinal, mesenterial as well as peripheral arteries. Typically, a rupture in the ascending aorta or aortic root may provoke a pericardial hematoma. The existence of a pericardial pericardial hematoma caused by an aortic rupture can dramatically reduce the chances of survival [1–4]. Accordingly, the duration from the onset of typical symptoms (e.g., acute tearing and migrating back pain) or atypical symptoms (e.g., dyspnea, syncope, stroke, leg pain or paraplegia) to an adequate treatment remains highly relevant, particularly in the case of a pericardial hematoma. To prevent cardiac decompensation, a rapid drainage of the hemorrhaged pericardial effusion and surgical treatment is required. Furthermore, the extent of an intervention in preoperatively decompensated patients should be evaluated.

There is limited published research on surgical outcomes for the high-risk group of AAAD patients with a pericardial hematoma. The aim of our present study was to evaluate the surgical procedure as well as the in-hospital and follow-up outcomes of this cohort operated on at our center.

## Methods

### Study population and study design

In this study, we included all patients that received surgical treatment between January 2000 and January 2018 (430 patients; 67% male; median age 64 years (interquartile range 54–71 years)) at our facility for acute aortic dissection DeBakey type I. Chronic dissections as well as DeBakey type II + III dissections were not included in the study. The subjects were divided in two groups. A pericardial pericardial hematoma occurred in 162 patients (37,67%; Group A) and in 268 patients without a pericardial hematoma (62,33%; Group B). Data were collected contemporaneously in our outpatient clinic or was actively collected by a study nurse team. Data were reviewed retrospectively and supplemented from the patients’ records after informed consent. Follow-up data was collected up until February 2022. This retrospective study was approved by the institutional ethics committee. Preoperative characteristics of the treated cohort are presented in Tables 1 and 2.

**Table 1:** Preoperative data

Characteristics	Entire cohort	Pericardial hematoma	Without pericardial hematoma	*P*-value
Total patients	n = 430	n = 162	n = 268	
Age at surgery (years), median (IQR)	63.7 (53.6–71.4)	64.3 (53.3–71.6)	63.5 (53.7–71.1)	0.510
Sex male, n (%)	289 (67.2)	108 (66.7)	181 (67.5)	0.852
BMI, median (IQR)	26.2 (24.2–29.1)	26.2 (24.4–29.2)	26.2 (24.1–28.4)	0.511
Hypertension, n (%)	278 (64.7)	106 (65.4)	172 (64.2)	0.792
Diabetes mellitus, n (%)	30 (7.0)	9 (5.6)	21 (7.8)	0.368
Pvod, n (%)	19 (4.4)	5 (3.1)	14 (5.2)	0.296
COPD, n (%)	40 (9.3)	11 (6.8)	29 (10.8)	0.163
Coronary heart disease, n (%)	46 (10.7)	15 (9.3)	31 (11.6)	0.453
Hyperthyreosis, n (%)	3 (0.7)	1 (0.6)	2 (0.7)	1.000
Hypothyreosis, n (%)	36 (8.4)	10 (6.2)	26 (9.7)	0.200
Artial firbillation, n (%)	53 (12.3)	26 (16.0)	27 (10.1)	0.068
Marfan syndrom, n (%)	19 (4.4)	4 (2.5)	15 (5.6)	0.126
Pericardial pericardial hematoma, n (%)	162 (37.7)	162 (100.0)	0 (0.0)	–
Bicuspid aortic valve, n (%)	21 (4.9)	7 (4.3)	14 (5.2)	0.674
Preoperative intubation, n (%)	55 (12.8)	32 (19.8)	23 (8.6)	**0.001**
Mechanical resuscitation, n (%)	38 (8.8)	34 (21.0)	4 (1.5)	** < 0.001**
Cardiac-reoperation, n (%)	15 (3.5)	3 (1.9)	12 (4.5)	0.150

Significance *P* < 0.05 are in bold

*BMI* Body mass index, *IQR* Interquartile range, *PVOD* Peripheral vascular occlusion disease, *COPD* Chronic obstructive occlusion disease)

**Table 2:** Preoperative data

Characteristics	Entire cohort	Pericardial hematoma	Without pericardial hematoma	*P*-value
Malperfusion, n (%)	135 (31.4)	57 (35.2)	78 (29.1)	0.188
Cerebral malperfusion, n (%)	49 (11.4)	18 (11.1)	31 (11.6)	0.885
Visceral malperfusion, n (%)	36 (8.4)	20 (12.3)	16 (6.0)	**0.021**
Renal malperfusion, n (%)	49 (11.4)	24 (14.8)	25 (9.3)	0.083
Limb malperfusion, n (%)	61 (14.2)	27 (16.7)	34 (12.7)	0.252
Hemiparese, n (%)	26 (6.0)	11 (6.8)	15 (5.6)	0.615
Paraparese, n (%)	15 (3.5)	9 (5.6)	6 (2.2)	0.069
Seizure, n (%)	7 (1.6)	5 (3.1)	2 (0.7)	0.109
Evidence of Stroke CT, n (%)	26 (6.0)	8 (4.9)	18 (6.7)	0.453
Neurologic symptoms, n (%)	84 (19.5)	33 (20.4)	51 (19.0)	0.734
Dissection supra-aortic arteries, n (%)	88 (20.5)	32 (19.8)	56 (20.9)	0.776
Dissection LCA, n (%)	12 (2.8)	5 (3.1)	7 (2.6)	0.770
Dissection RCA, n (%)	42 (9.8)	17 (10.5)	25 (9.3)	0.693
Iatrogenic dissection, n (%)	11 (2.6)	0 (0.0)	11 (4.1)	**0.008**
Onset of pain to surgery time (h), median (IQR)	6.0 (4.0–12.1)	6.0 (4.0–12.0)	7.0 (4.0–12.9)	0.696

Significance *P* < 0.05 are in bold

*LCA* Left coronary artery, *RCA* Right coronary artery, *CT* Computer tomography

### Follow–up

The clinical follow–up ended in August 2021 and was 100% complete. We received informed consent from patients to collect follow-up data. Patients were regularly seen in our outpatient clinic. In addition, CTA or MRI examinations were performed at fixed intervals.

### Definitions

**Fig. 1:**
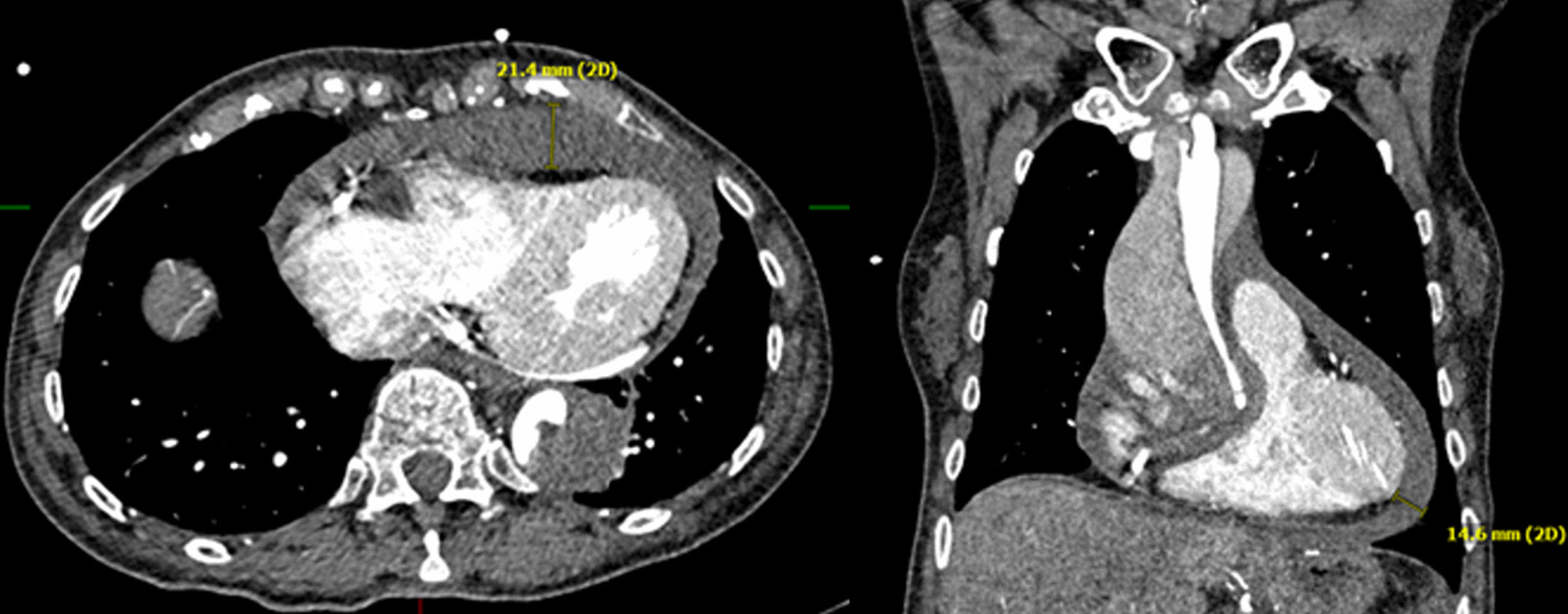
Transversal (left) and coronal (right) CT scan with imaging of pericardial hematoma due to AAAD

The diagnosis of a pericardial hematoma was based on the radiological evidence of a hemopericardium using CT, MRT or echocardiography. Due to the fact that not every patient received an echocardiographic examination, CT findings with bloody pericardial effusion > 1 cm were included (Fig. 1). The detected pericardial hematoma (> 1 cm) had to be located next to the right and/or left ventricle. Pericardial effusion had to be clarified as “bloody”. Patients with serous pericardial effusion were not included in the pericardial hematoma group. Malperfusion was defined as an occlusion or a false lumen perfusion of one relevant artery per organ. Furthermore, the diagnosis of malperfusion was defined according to the classification of Sievers et al. [5]. Stages M2 and M3 ((−), ( +)) were assigned to the malperfusion group. M2, dissection of at least 1 supra-aortic vessel or aortic arch true lumen collapse with (M2+) or without (M2−) clinical symptoms of cerebral (stroke) or upper extremity (pulse deficit, pain, pallor, paresthesia) malperfusion; M3, dissection or false lumen origin of at least one visceral, renal or one iliac artery or aortic true lumen collapse entailing functional closure of at least one visceral, renal or iliac artery offspring, with (M3+) or without (M3−) clinical symptoms [5].

For this, the patient`s CT scans were analyzed. Neurological symptoms were defined as the preoperative existence of a neurological disability with or without early-stage evidence of stroke using radiologic imaging. Patients that preoperatively presented severe neurologic symptoms, such as hemiplegia, dysarthria or apraxia without a performed cerebral CT scan with postoperative evidence of stroke were assigned to the preoperative stroke group. A postoperative stroke was confirmed by CT or magnetic resonance imaging. Dissections induced during open heart surgery were specified as iatrogenic dissections. Dissections of the coronary arteries were intraoperatively visible or preoperatively discovered during coronary angiography. The neurological symptoms were assessed by the emergency medicine team, anaesthesiologist and surgeon performing the operation, before strong sedative or narcotic medication was initiated.

One unit of erythrocyte concentrates, fresh frozen plasma or platelet concentrates is equal to 250 ml, respectively.

### Perioperative management and surgical technique

According to our standardized protocol, all patients with acute AAAD are transferred to the operation theater promptly after the initial diagnosis. To avoid early decompensation, intubation is not performed before preoperative preparations are completed. After intubation and median sternotomy, extracorporeal circulation (ECC) is performed. The technique for cannulating the ascending aorta, also in AAAD, was previously published by our group [6, 7]. We perform a direct cannulation of the ascending aorta after identifying the true lumen using trans esophageal echocardiography. The left side of the heart is vented through the right superior pulmonary vein. The aorta is clamped, also in AAAD patients. Cardioplegia is administered directly into the coronary ostia. Blood cardioplegia is our preferred method of myocardial protection. In our department, moderate hypothermic circulatory arrest is established in partial as well as total arch repair. During this time, the patient is cooled to a nasopharyngeal temperature of 22–26 °C and the aortic root/ascending aortic procedure is performed. Other concomitant procedures (e.g., CABG) are also performed if necessary. The cardioplegia is repeated approximately every 30 min [7]. In all cases, either a proximal, subtotal (involving the replacement of the brachiocaephalic trunk) or total arch replacement with ET or FET, hypothermic circulatory arrest (temperatures between 22 and 26 °C) and bilateral selective antegrade cerebral perfusion was performed. The application of SACP varied when a limited arch repair was performed. In 2010 we started applying the beating heart technique for cardio-protection during total arch repair [8].

Due to the long periods covered in this study, the surgical technique regarding the choice of aortic grafts evolved significantly.

#### Extended arch repair

During the period from 2000 to 2010, the FET technique was performed using a custom-made Chavan–Haverich prosthesis followed by a prefabricated Chavan-Haverich hybrid graft [9, 10] (Curative GmbH, Dresden, Germany). Furthermore, we used the Jotec E-vita hybrid graft 2005 until 2010 [11]. The island technique (en bloc) was performed to reattach the supraaortic vessels until 2010. In cooperation with Vascutek Terumo (Terumo^®^, Glosgow, UK), the four-branched frozen elephant trunk (FET) that we used continuously from 2010 was developed [12, 13]. In 2007, for a total or hemiarch replacement, we changed our strategy from a straight graft with island technique to the branched Sienna™ graft (Terumo^®^, Glosgow, UK). The extensive use of a branched aortic arch prosthesis resulted in major technical changes. As a consequence of these changes, the arch replacement was performed after completing the cardiac and distal aortic repair. Head vessels were anastomosed to the corresponding side branches of the graft at the end of the procedure [12].

#### Proximal arch repair

An isolated replacement of the proximal aortic arch was performed using different straight Dacron grafts.

### Statistical analysis

SPSS 27 Statistics software (IBM Corp. Released 2020; IBM SPSS Statistics for Windows, Version 27.0; Armonk, NY: IBM Corp.) was used for the data analysis. A normal distribution of variables was calculated using the Kolmogorov–Smirnov test. Categorical variables were given as absolute numbers (n) and proportions. Normally distributed continuous variables were given as mean ± standard deviation, while continuous variables without normal distribution were given as median and interquartile ranges (IQR). Fisher’s exact test was used to detect differences in the categorical variables. Differences in the continuous variables were tested using the Mann Whitney U test. Kaplan–Meier analysis and log-rank were used for the evaluation of survival, and the log-rank test was used to test for differences. We did not correct for multiple testing. A univariable analysis was performed to test for any association between the variables and in-hospital mortality.

## Results

### Preoperative patient characteristics

The preoperative patient characteristics are given in Tables 1 and 2. No significant differences were found between the patients regarding age (A: 63.7 years (53.6–71.4); B: 63.5 years (53.0–71.1); *P*: 0.510) and BMI (A: 26.2 (24.4–29.2); B: 26.2 (24.1–28.4); *P*: 0.511). The majority of the cohort were male patients in both groups (A: n = 108 (66.7%); B: n = 181 (67.5%); *P*: 0.852). Hypertension occurred in 65.4% (n = 106) of the patients with hematoma and in 64.2% (n = 172) of those without (*P*: 0.972). Concomitant diseases like PVOD, COPD and coronary heart disease were fairly equally distributed in both cohorts. Atrial fibrillation was increasingly observed in group A (A: 26% (n = 26.0); B: 10.1% (n = 27); *P*: 0.068). It was notable that patients with a preoperative bloody pericardial effusion had a significant requirement for mechanical resuscitation (A: 21% (n = 34); B: 1.5% (n = 4); *P*: < 0.001) and were more often admitted to the operation theater in an intubated status (A: 19.8% (n = 32); B: 8.6% (n = 23); *P*: 0.001). The incidence of visceral malperfusion differed significantly between both groups, showing a higher rate in the cohort with pericardial hematoma (A: 11.7% (n = 19); B: 6% (n = 16); *P*: 0.034). No relevant differences were detected in terms of neurological disabilities.

### Intraoperative data

Detailed intraoperative data are shown in Table 3. Patients with a preoperative pericardial hematoma had a shorter total operation time (A: 317.5 min (IQR: 260.5–386.5); B: 335.0 min (IQR: 259.3–409.8); *P*: 0.379) and SACP (selective antegrade cerebral protection) (A: 28.5 min (IQR: 19.0– 60.0); B: 38.5 min (IQR: 20.0–80.0); *P*: 0.077). The median number of infused erythrocyte concentrates was moderately but significantly higher in the group with a preoperative pericardial hematoma (A: n = 7.0 (IQR: 4.0–12); B: n = 6.0 (IQR: 3.0–9.0); *P*: < 0.001). There was a significant difference between groups in the intraoperative use of fresh frozen plasma concentrates (A: n = 6 (IQR: 6.0–10.0); B: n = 6 (IQR); *P*: 0.10). The beating heart procedure was performed significantly more often in Group B (A: 8.0 (4.9%); B: 58.0 (21.6%); *P*: < 0.001). Significant differences were detected regarding the operative procedure. Whereas Group A had more proximal arch replacement procedures (A n = 84 (51.9%); B: n = 108 (40.3%); *P*: 0.020), the cohort without the bloddy pericardial hematoma was relevantly more often treated with total arch replacement using a frozen elephant prosthesis (A: n = 29 (17.9%); B: n = 92 (34.3%); *P*: < 0.001). A Yacoub procedure was performed significantly more often in patients with a pericardial hematoma (A: n = 13 (8.0%); B: n = 6 (2.2%); *P*: 0.005). A minority of 12 patients received a Florida sleeve procedure. The incidence of intraoperative deaths was elevated in patients with a preoperative existing pericardial hematoma (A: n = 10 (6.2%); B: n = 2 (0.7%); *P*: 0.001.

**Table 3:** Detailed intraoperative data

Characteristics	Entire cohort	Pericardial hematoma	Without pericardial hematoma	*P*-value
Total patients	n = 430	n = 162	n = 268	
Total operation time (min), median (IQR)	330.5 (259.8–404.3)	317.5 (260.5–386.5)	335.0 (259.3–409.8)	0.379
Cardiopulmonary bypass time (min), median (IQR)	217.0 (169.5–285.0)	216.0 (177.5–270.3)	217.5 (165.0–287.8)	0.960
Aortic cross-clamp time (min), median (IQR)	126.0 (92.8–161.3)	129.0 (102.8–162.0)	121.5 (88.0–159.5)	0.109
HCA (hypothermic circulatory arrest) time (min), median (IQR)	36.0 (25.0–52.0)	36.0 (26.8–52.0)	35.0 (24.0–52.0)	0.353
SACP (Selective antegrade cerebral perfusion) time (min), median (IQR)	32.5 (19.0–76.0)	28.5 (19.0–60.0)	38.5 (20.0–80.0)	0.077
Minimal core temperature (C°), median (IQR)	24.7 (22.2–26.0)	25.0 (22.8–26.0)	24.3 (22.0–26.0)	0.066
Erythrocyte concentrates, median (IQR)	6.0 (4.0–10.0)	7.0 (4.0–12.0)	6.0 (3.0–9.0)	0.001
Fresh frozen plasma, median (IQR)	6.0 (4.0–10.0)	6.0 (6.0–10.0)	6.0 (4.0–8.0)	0.010
Platelet concentraltes, median (IQR)	3.0 (2.0–4.0)	2.0 (2.0–4.0)	3.0 (2.0–4.0)	0.957
Arch replacement				
Proximal arch replacement, n (%)	192 (44.7)	84 (51.9)	108 (40.3)	0.020
Subtotal arch replacement, n (%)	34 (7.9)	13 (8.0)	21 (7.8)	0.944
Total Arch replacement, n (%)	36 (8.4)	17 (10.5)	19 (7.1)	0.217
Total Arch replacement Elephant trunk, n (%)	47 (10.9)	19 (11.7)	28 (10.4)	0.680
Total Arch replacement Frozen Elephant trunk, n (%)	121 (28.1)	29 (17.9)	92 (34.3)	< .001
Bio glue, n (%)	146 (34.0)	45 (27.8)	101 (37.7)	0.036
Aortic valve replacement				
Biologic, n (%)	65 (15.1)	25 (15.4)	40 (14.9)	0.887
Mechanic, n (%)	67 (15.6)	30 (18.5)	37 (13.8)	0.192
Root involvement, n (%)	258 (60.0)	106 (65.4)	152 (56.7)	0.074
Bentall, n (%)	129 (30.0)	54 (33.3)	75 (28.0)	0.241
David, n (%)	98 (22.8)	35 (21.6)	63 (23.5)	0.649
Yacoub, n (%)	19 (4.4)	13 (8.0)	6 (2.2)	0.005
CABG, n (%)	77 (17.9)	26 (16.0)	51 (19.0)	0.435
ECMO, n (%)	19 (4.4)	8 (4.9)	11 (4.1)	0.684
Exitus in tabula, n (%)	12 (2.8)	10 (6.2)	2 (0.7)	0.001

*HCA* Hypothermic circulatory arrest, *SACP* Selective antegrade cerebral perfusion, *CABG* Coronary artery bypass graft, *ECMO* Extracorporeal membrane oxygenation

### Postoperative data

**Fig. 2:**
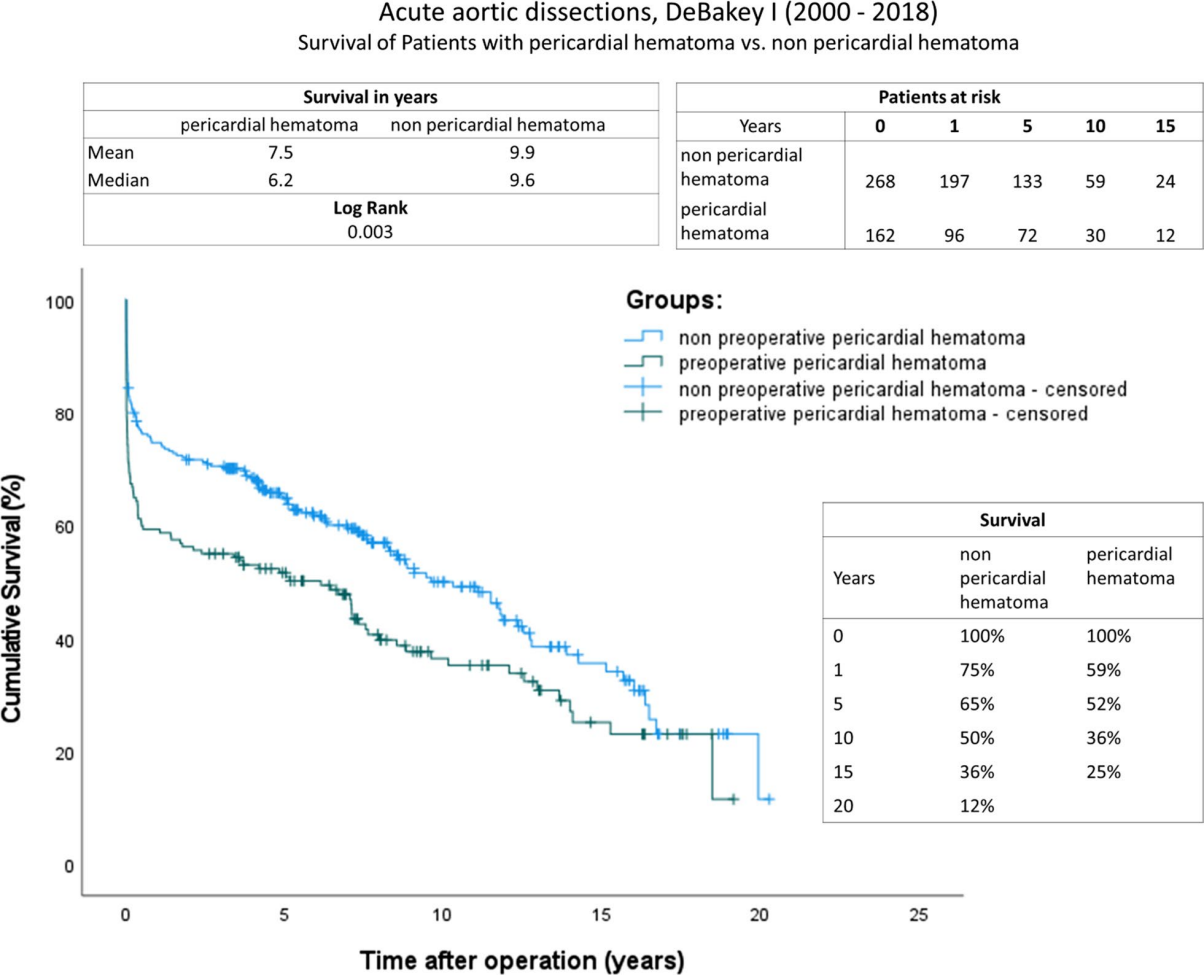
Kaplan–Meier curves showing survival with and without preoperative pericardial hematoma. The x-axis denotes the time after operation

The postoperative data are summarized in Table 4. Survival time (A: d = 1339.0 (12.8–2948.8); B: d = 1798.5 (196.5–3296.0); *P*: 0.012) as well as 30-day mortality (A: n = 48 (29.6%); B: n = 46 (13.8%); *P*: 0.002) differed significantly between the groups. The respective Kaplan survival curves are shown in Fig. 2. We found significant survival differences with a mean survival of 7.5 years in the pericardial hematoma group and 9.9 years in the group without the pericardial hematoma (log rank, *P*: 0.003). The duration of the ICU treatment was moderately decreased in the pericardial hematoma group (A: d = 4.0 (2.0–7.0); B: d = 4.5 (2.0–9.0); *P*: 0.24). Furthermore, the rate of re-thoracotomy was elevated in Group A (A: n = 34 (21.0%); B: n = 37 (18.8%); *P*: 0.052). However, no significant differences were detected regarding ventilation time, dialysis requirement and postoperative stroke.

**Table 4:** Postoperative data

Characteristics	Entire cohort	Pericardial hematoma	Without pericardial hematoma	*P*-value
Total patients	n = 430	n = 162	n = 268	
Survival time (days), median (IQR)	1667.5 (71.0–3212.0)	1339.0 (12.8–2948.8)	1798.5 (196.5–3296.0)	**0.012**
Ventilation time (h)	48.0 (21.0–138.3)	47.0 (18.0–113.0)	48.5 (22.0–145.5)	0.206
Intensive care unit (days), median (IQR)	4.0 (2.0–8.0)	4.0 (2.0–7.0)	4.5 (2.0–9.0)	**0.024**
Rethoracotomy, n (%)	71 (16.5)	34 (21.0)	37 (13.8)	0.052
Dialysis, n (%)	55 (12.8)	21 (13.0)	34 (12.7)	0.934
30 days mortality, n (%)	94 (21.9)	48 (29.6)	46 (17.2)	**0.002**
CCT stroke, n (%)	84 (19.5)	29 (17.9)	55 (20.5)	0.506
New-onset stroke, n (%)	37 (8.6)	13 (8.0)	24 (9.0)	0.739
Persisting cerebral malperfusion, n (%)	16 (3.7)	5 (3.1)	11 (4.1)	0.589
Persisting limb malperfusion, n (%)	13 (3.0)	4 (2.5)	9 (3.4)	0.774
Persistining visceral malperfusion, n (%)	10 (2.3)	1 (0.6)	9 (3.4)	0.098
Persisting renal malperfusion, n (%)	20 (4.7)	9 (5.6)	11 (4.1)	0.489

Significance *P* < 0.05 are in bold

*CCT* Cranial computer tomography

### Long-term outcome

Follow-up data are listed in Table 5. There was a higher demand for secondary aortic surgery in the group without the existing pericardial hematoma (A: 8.6%; B: 15.7%; *P*: 0.036). The retrospective Kaplan–Meier survival curves are shown in Fig. 2. We found significant survival differences with a mean survival of 7.5 years. In the pericardial hematoma group and 9.9 in the group without the pericardial hematoma (log rank, *P*: 0.003).

**Table 5:** Follow-up data

Characteristics	Entire cohort	Pericardial hematoma	Without pericardial hematoma	*P*-value
Total patients	n = 430	n = 162	n = 268	
Secondary aortic operation, n (%)	53 (12.3)	12 (7.4)	41 (15.3)	**0.016**
Re-operation identical area, n (%)	16 (3.7)	3 (1.9)	13 (4.9)	0.111
Re-operation downstream aorta, n (%)	37 (8.6)	9 (5.6)	28 (10.4)	0.080
TAA repair, n (%)	9 (2.1)	3 (1.9)	6 (2.2)	1.000
Y prothesis, n (%)	4 (0.9)	2 (1.2)	2 (0.7)	0.634
Descending repair, n (%)	18 (4.2)	5 (3.1)	13 (4.9)	0.376
Hybrid, n (%)	7 (1.6)	2 (1.2)	5 (1.9)	0.715
TEVAR, n (%)	13 (3.0)	2 (1.2)	11 (4.1)	0.144
EVAR, n (%)	5 (1.2)	1 (0.6)	4 (1.5)	0.654
Aortic fenestration (%)	2 (0.5)	0 (0.0)	2 (0.7)	0.529

Significance *P* < 0.05 are in bold

*TAA* Open thoracic aneurysm aortic repair, *TEVAR* Thoracic endovascular aortic repair, *EVAR* Abdominal endovascular aneurysm repair

**Fig. 3:**
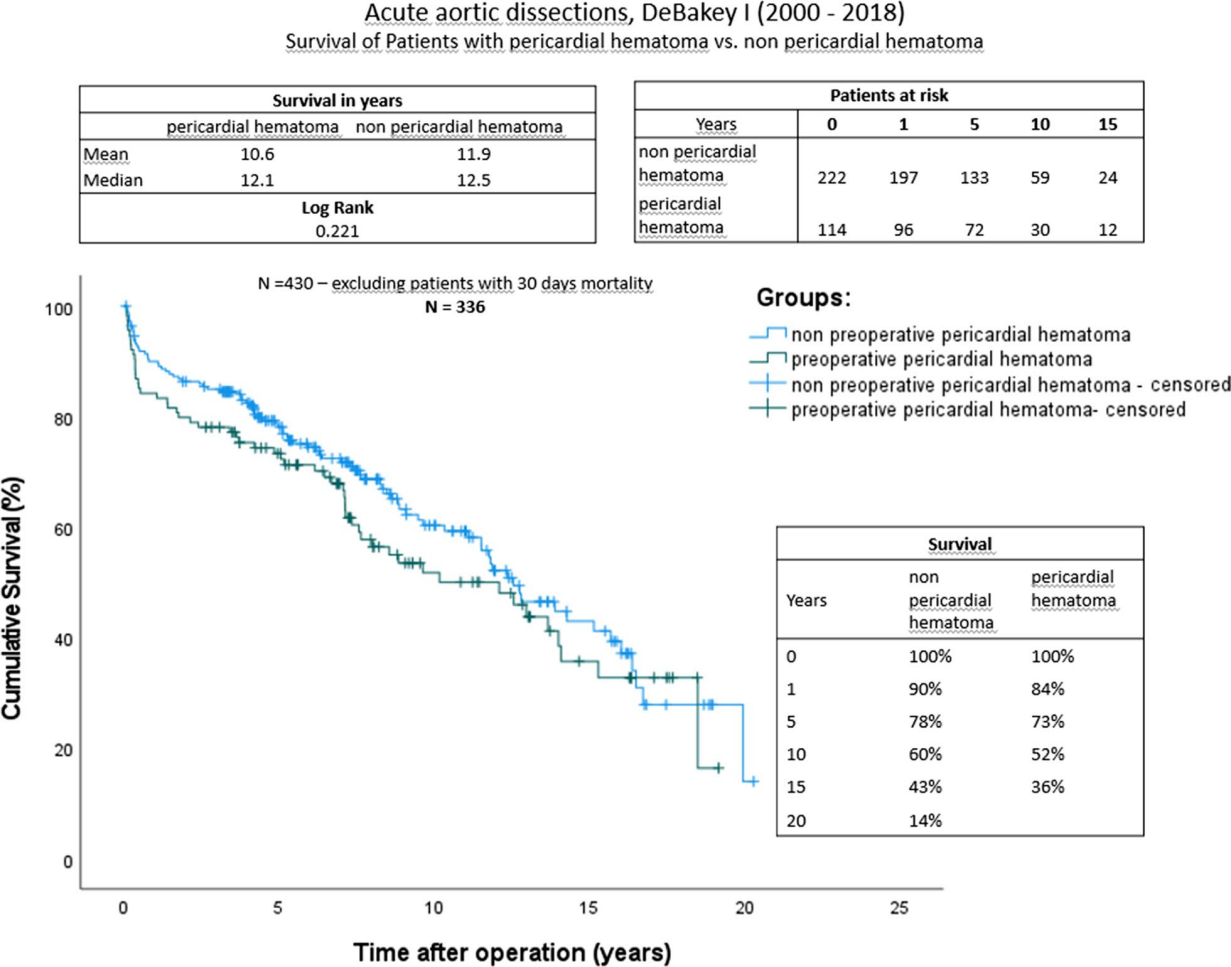
Kaplan–Meier curves showing survival with and without preoperative pericardial hematoma after excluding to 30 day mortality cohort. The x-axis denotes the time after operation

Long-term survival was influenced by the immense early mortality of group A. No significant was detected after excluding (Fig. 3). The survival of both groups was comparable approximately 1 year after surgery.

## Discussion

An acute aortic dissection is a life-threatening disease. In the present study, we assessed the consequences of a preoperative pericardial pericardial hematoma in cases of AAAD. Recent data from the International Registry of Acute Aortic Dissections (IRAD) identified the prevalence of a pericardial hematoma as a risk factor for early mortality [14]. However, is there a consequence in terms of an adjustment to the surgical procedure due to the existence of this known risk factor? Furthermore, does a patient’s hemodynamic instability require a limited aortic arch repair?

According to our policy, in patients with limited conditions like cardiogenic shock, preoperative coma or malperfusion, a proximal arch repair is favored to reduce intervention time and to ensure rapid reperfusion. Patients without severe clinical symptoms, young patients, Marfan patients or patients presenting a true lumen collapse should be evaluated for extend arch repair. Nevertheless, the cohort of AADA patients remains heterogenic.

The patient’s status on admission differed significantly between the two groups. The incidence of preoperative intubation and mechanical resuscitation was higher in patients with a pericardial pericardial hematoma, as evidence of instable hemodynamic preconditions. Despite previous publications [15] that describe patients with a pericardial hematoma that could not even survive the transfer to hospital, a high number (n = 162) of patients with AAAD with a pericardial hematoma received surgical treatment at our hospital. Although the onset of pain to surgery time did not differ significantly (A: 6.0 (4.0–12.0); B: 7.0 (4.0–12.9); *P*: 0.696). It was overall reduced in comparison to the IRAD data, which had a median time of 8.3 h from emergency department presentation and intervention[16]. Neurological symptoms correlated with dissection of the supraaortic arteries. These symptoms are due to general hypotension or dissection/occlusion of one or more aortic side branches supplying brain, spinal cord or peripheral nerves.

A high number of full root replacement was detected (A: 65.4%; B: 56.7%; *P*: 0.074) in our study. Our group has previously evaluated the extent of the root procedure in AADA patients. We concluded that full root replacement does not increase the perioperative risk in patients who undergo frozen elephant trunk for acute dissection. In this cohort, carful patient selection is important for these complex procedures [17].

Nevertheless, this compromised cohort fulfilled further risk factors for early mortality [18], including preoperative intubation (A: 19.8%; B: 8.6%; *P*: 0.001) and mechanical resuscitation (A: 21%; B: 1.5%; *P*: < 0.001).

An increasing chance of pre-existing atrial fibrillation was observed in patients with bloody pericardial effusion (A: 16.0%; B: 10.1%; *P*: 0.068). This circumstance may be explained by local cardiac congestion due to the pericardial hematoma. Furthermore, we observed visceral malperfusion to be more likely in patients with pericardial effusion (A: 12.3%; B: 6.0%; *P*: 0.034). Malperfusion is also known to be an independent risk factor for early death [19]. In addition, a patient’s low output syndrome in cases of pericardial hematoma may promote further true lumen collapse due to lower blood pressure. The current literature still requires an answer to whether limited or extended arch repair in this compromised cohort is preferred. Limited aortic arch repair was preferred in patients with the pericardial hematoma (proximal arch replacement: A: 51.9%; B: 40.3%; *P*: 0.020; FET: A: 17.9%; B: 34.3%; *P*: 0.001). In contrast to these results, it may be reasonable that relevant malperfusion requires prompt extended aortic arch treatment. Previous publications by Kazui et al. [20] describe how extended aortic arch repair could be applied without increasing the patient’s perioperative risk.

While our mortality of patients without a pericardial hematoma (17.2%) coincidences with the German Registry for Acute Aortic Dissection Type A (16.9%) [21], 30-day mortality was significantly (*P*: 0.002) increased in the vulnerable cohort of Group A (29.6%). 30-day mortality was significantly increased in the pericardial hematoma group when preoperative intubation (A: 31.3% vs. B: 13%), mechanical resuscitation (A: 37.5% vs. B: 2.2%; *P*: < 0.001) or malperfusion (A: 68.8%; vs B: 47.8%; *P*: 0.040) occurred.

This has to result in a re-evaluation of the surgical decision-making process. Existing options include a diversification of the preoperative treatment. Three options must be evaluated: preoperative pericardial drainage, conservative treatment or extended arch repair. The number of patients that received preoperative mechanical resuscitation was 21% (vs. 1.5% without pericardial hematoma). Recent studies have concluded that initial emergency pericardial drainage without aortic repair was associated with favorable early and midterm outcomes. In correlation with the increased demand for resuscitation, the placement of pericardial drainage should be evaluated. In particular, prior to transfer to the aortic center, a pericardial drainage may prevent preoperative death [22, 23]. Likewise, a conservative treatment in a selected cohort may present a reasonable option. Existing studies have also proven that the surgical approach did not achieve a significant survival advantage over conservative treatment in choice for older patients [1, 14, 24–28]. Nevertheless, our study reveals that even extended aortic surgery might contribute substantially to a favorable outcome in a selective cohort. For instance, patients with advances malperfusion might benefit from more aggressive treatment. Careful patient selection and surgical experiences remain important factors in such complex procedures.

According to our current study, the preoperative clinical conditions for patients with a pericardial hematoma predict the poor outcome.

## Limitations

Due to the fact that this is a retrospective study, it carries all the potential risks and biases associated with studies of this nature. Furthermore, the final decision regarding the surgical procedure was made by the surgeon. Between the years 2000–2018, a total of 25 surgeons performed the operative treatment of the patients. It is assumed that surgical skill levels varied. In addition, for the diagnosis of a low cardiac output syndrome, the implementation of a preoperative echocardiography with documentation of the ejection fraction is a mandatory approach. A patient’s outcome is undeniably associated with the factor of time. A large number of patients dying prior to admission can be expected.

## Conclusions

A pericardial hematoma in cases of AAAD remains a life-threatening constellation. This study shows that even the existence of a pericardial hematoma significantly limits the chances of survival. Patients in this cohort were significantly compromised regarding their preoperative conditions. We have proven that cardiac compression is associated with preoperative intubation and mechanical resuscitation. Nevertheless, this vulnerable cohort remains heterogenic. Preoperative and surgical treatment has to be individually adjusted. Patients with pericardial hematoma must be further evaluated for preoperative pericardial drainage. In the event of long transfer times to an aortic center a slow drainage should be discussed to prevent early mortality.

In summary, a relevant number of patients with a pericardial pericardial hematoma in cases of AAAD present themselves in a lethal condition. According to our data, based on the surgeon’s decision, a limited aortic repair is preferred in these compromised cases. Nonetheless, further studies will be necessary to investigate the treatment of these high-urgency patients.

## Data Availability

Data were collected in our outpatient clinic. Data were reviewed retrospectively and supplemented from the patient’s records after informed consent.

## Abbreviations

AAAD: Acute type A aortic dissection
BMI: Body mass index
IQR: Interquartile range
PVOD: Peripheral vascular occlusion disease
COPD: Chronic obstructive occlusion disease
FET: Frozen elephant trunk
SACP: Selective antegrade cerebral protection
PVOD: Peripheral vascular occlusion disease
COPD: Chronic obstructive pulmonary disease
IQR: Interquartile ranges
CCT: Cranial computer tomography
TAA: Thoracic aneurysm aortic repair
TEVAR: Thoracic endovascular aortic repair
EVAR: Endovascular aortic repair
IRAD: International Registry of Acute Aortic Dissections
HCA: Hypothermic circulatory arrest
SACP: Selective antegrade cerebral perfusion
CABG: Coronary artery bypass graft
ECMO: Extracorporeal membrane oxygenation)
LCA: Left coronary artery
RCA: Right coronary artery
CT: Computer tomography
