# Correction: Preoperative pericardial hematoma in patients with acute type A aortic dissection (AAAD): Do we need an adjusted treatment?

**DOI:** 10.1186/s13019-023-02179-4

**Published:** 2023-03-14

**Authors:** Tim Kaufeld, Erik Beckmann, Linda Rudolph, Heike Krüger, Ruslan Natanov, Morsi Arar, Wilhelm Korte, Tobias Schilling, Axel Haverich, Andreas Martens, Malakh Shrestha

**Affiliations:** 1 Department of Cardiothoracic, Transplant and Vascular Surgery, Hanover Medical School, Carl – Neuberg Str.1, 30625 Hannover, Germany; 2 Minneapolis Heart Institute, Abbott Northwester Hospital, 920E 28th St., Minneapolis, MN 55417 USA; 3 Cardiovascular Medicine, Mayo Clinic, 200 First St. SW, Rochester, MN 55905 USA

**Correction to: Journal of Cardiothoracic Surgery (2023) 18:67** https://doi.org/10.1186/s13019-023-02152-1

Following publication of the original article [1], in this article the first name and the sur name of all the authors were interchanged and it should read as follow:

Tim Kaufeld^1*^, Erik Beckmann^1,2^, Linda Rudolph^1^, Heike Krüger^1^, Ruslan Natanov^1^, Morsi Arar^1^, Wilhelm Korte^1^, Tobias Schilling^1^, Axel Haverich^1^, Andreas Martens^1†^ and Malakh Shrestha^1,3†^

The original article has been corrected.
